# Relations Between Class Competition and Primary School Students’ Academic Achievement: Learning Anxiety and Learning Engagement as Mediators

**DOI:** 10.3389/fpsyg.2022.775213

**Published:** 2022-02-23

**Authors:** Guoqiang Li, Zhiyuan Li, Xinyue Wu, Rui Zhen

**Affiliations:** Jing Hengyi School of Education, Hangzhou Normal University, Hangzhou, China

**Keywords:** primary school students, class competition, academic achievement, learning anxiety, learning engagement, mediating role

## Abstract

This study aimed to analyze the relations between class competition and primary school students’ academic achievement, considering the possible mediating roles of learning anxiety and learning engagement. Participants were 1,479 primary school students from four primary schools in Zhejiang, China. We analyzed participants’ scores for class competition, learning anxiety, and learning engagement and their last two final exam scores. Class competition did not directly predict academic achievement, but indirectly affected academic achievement through learning anxiety and learning engagement. There were three effect paths: (1) class competition negatively predicted academic achievement by increasing learning anxiety; (2) class competition positively predicted academic achievement by promoting learning engagement; and (3) class competition affected academic achievement through multiple mediating effects of learning anxiety and learning engagement. This study highlights the important roles of learning anxiety and learning engagement in class competition and academic achievement, which have theoretical and practical significance.

## Introduction

Competition is a ubiquitous and age-old behavior pattern (Sun et al., 2015), and has become a prevalent problem across countries, cultures, and ethnic groups (Wong et al., 2006). For decades, psychological research has valued the study of competition (Sun et al., 2015). In the field of education, due to the limited high-quality educational resources, plus the driving force of students’ life goals, competition has also become a reality that students have to face (Posselt and Lipson, 2016). The primary school stage is the beginning of a person’s academic career, and it is also the basis of subsequent learning and future development. Parents often pay much attention to students’ academic achievement since children commence primary school life. This is especially reflected in students from some Asian countries such as China (Deng and Zou, 2015). As a result, primary school students may be unknowingly faced with academic competition. Children spend most of their time at school, which, after family, is the most important environment affecting child development (Volk et al., 2015). Because primary school students spend most of their school time in classes, academic competition among students is mainly reflected in class competition (Hu, 2018).

Academic achievement is a key indicator of students’ learning conditions, and provides an important basis for determining whether students can enter higher education. In addition, academic achievement is commonly used as a standard to judge the quality of school teaching. Therefore, students’ academic achievement receives substantial attention from students, parents and society. Various researchers have proposed that class competition is a critical factor in the education process (John et al., 2003; Ogbuehi and Fraser, 2007), and can have a negative impact on students’ academic achievement (Johnson and Johnson, 1994; Ames and Archer, 1988). The pressure perceived by students increases as class competition increases, and many children experience doubt regarding their learning abilities (Sommet et al., 2013). In addition, competitive instruction has been observed to stress the acquisition of low-level information rather than high-level ideas (Sullivan, 1980), which can have a negative impact on children’s academic achievement. However, previous studies have reported conflicting results. In some studies, individual competition was reported to have a positive impact on students’ academic achievement (Fisher, 1976; Tauer and Harackiewicz, 2004). He et al. (2011) reported a significant positive correlation between class competition and academic achievement. Competition can be divided into benign competition and vicious competition (Horney, 1937; Ryckman et al., 1990), and some researchers (Burguillo, 2010; Olitsky, 2011) have analyzed class competition from a dialectical perspective, and reported that benign competition stimulated students’ motivation, promoted individuals to learn from each other, and caused individuals to perform better, but vicious competition resulted in inaccurate cognition, children’s loss of correct judgment of their own value, and limit collaboration among students, hinder their progress, and ultimately have negative effects on their academic achievement. However, other researchers have reported that competition has no clear effect on students’ academic achievement (Pesout and Nietfeld, 2021) and that class competition cannot predict academic achievement in students (Pang, 2009). Thus, the relation between class competition and academic achievement is complex. Consequently, the effect of class competition on students’ academic achievement remains controversial, and its internal mechanisms require further investigation.

### Mediating Role of Learning Anxiety

Learning anxiety may be an important factor in the negative relation between class competition and academic achievement. From a psychological perspective, class competition reflects students’ and teachers’ perceptions regarding the competitive atmosphere in the class or classroom environment (Fraser, 1986). Therefore, class competition is the type of psychological environment that students perceive. Learning anxiety refers to uneasy or unpleasant psychological reflection caused by internal conflict and is a specific state of tension in a student group (Woodman and Hardy, 2001). Learning anxiety is therefore a negative emotion produced by students in the process of learning (Pekrun, 2005).

Relative deprivation theory suggests that students tend to evaluate their academic level based on comparison with other students in the same class (Davis, 1966). Because of differences in learning ability among students, it is inevitable that some students win while other students fail in a competitive situation. Anxiety is triggered when students anticipate that they may fail (Pekrun, 2006). When the level of class competition is low, the pressure caused by students comparing themselves with each other is low, and students learn easily and happily. However, when class competition exceeds a certain level, students’ learning pressure increases and they may begin to doubt themselves, which results in negative emotions such as high anxiety (Johnson and Johnson, 1994). Therefore, it follows that class competition may increase students’ learning anxiety (Epstein and Harackiewicz, 1992; Quach et al., 2015).

Previous studies confirmed that learning anxiety can lead to poor academic achievement among students (Sariem et al., 2014; Chang and Beilock, 2016; D’Agostino et al., 2021). Emotions have been found to affect a wide range of cognitive processes, including attention, memory storage and retrieval, social judgment, decision making and cognitive problem solving (Clore and Huntsinger, 2009). Students in a state of learning anxiety will exhibit task-irrelevant thinking and a reduction in the cognitive resources available for task purposes, as well as becoming distracted during learning, causing low learning efficiency and a decline in academic achievement (Pekrun et al., 2017). Some researchers have examined the relation between learning anxiety and academic achievement in specific subjects, particularly mathematics. Previous studies reported that mathematics anxiety negatively predicted mathematics achievement (Foley et al., 2017; Wang et al., 2019; Tomasetto et al., 2021). Mathematics anxiety may impair performance by diverting cognitive resources from task-relevant purposes (i.e., a mathematics task) to task-irrelevant aspects (e.g., worry) (Ching, 2017; Ching et al., 2020). Further research revealed that, compared with boys, girls have less fun and more anxiety in mathematics learning (Pekrun and Stephens, 2010; Dowker et al., 2019).

### Mediating Role of Learning Engagement

A possible reason for the positive correlation between class competition and academic achievement is the mediating role of learning engagement. Learning engagement is considered to be a positive, fulfilling, and work-related state of mind that is characterized by vigor, dedication, and absorption (Schaufeli et al., 2002b). Vigor is characterized by high levels of energy and mental resilience while working and by the willingness and ability to invest effort in one’s work. Dedication is characterized by a sense of significance, enthusiasm, inspiration, pride, and challenge. Absorption is characterized by being fully concentrated and happily engrossed in one’s work (Schaufeli et al., 2002a).

Class competition may promote learning engagement (Tauer and Harackiewicz, 2004; Reyes et al., 2012) and enhance students’ pressure and motivation in learning. Students will consciously invest in learning to ensure or improve their status among their classmates (Roth et al., 2009). Social learning theory (Bandura, 1978) posits that when many students in a class work hard, they serve as role models with which other students will compare themselves and imitate, promoting their engagement in learning activities (Hasan and Bagde, 2013). Learning engagement is positively correlated with academic achievement (Schaufeli et al., 2002a; Chang and Beilock, 2016). When the level of learning engagement is high, students will actively invest more time and energy in learning activities, and they will also have positive emotional experiences in learning (Schaufeli and Bakker, 2004; Liu et al., 2020), which plays an important role in promoting students’ academic achievement (Pietarinen et al., 2014; Bayoumy and Alsayed, 2021).

### Relation Between Learning Anxiety and Learning Engagement

Learning anxiety, as a negative emotion in an academic context, may have an adverse effect on students’ learning engagement. Studies have shown that students’ emotions are critical to their willingness to learn and their volitional control over the learning process (Pekrun, 2005). Positive emotions can stimulate learners’ motivation and promote high engagement in learning (Frenzel et al., 2007; Wara et al., 2018), whereas negative emotions (e.g., anxiety) reduce learning efficiency and may even cause serious learning delays (Macher et al., 2012). In addition, learning anxiety means students focus more on threats and failures, which restricts their cognition and activity maps (Fredrickson, 2001) and occupies their limited cognitive resources (Pekrun and Stephens, 2010). In turn, this affects students’ processing of current learning tasks (Owens et al., 2012). When students fail to meet the cognitive requirements of academic activities, they gradually develop low learning engagement (Zhen et al., 2017).

### Present Study and Hypotheses

**FIGURE 1:**
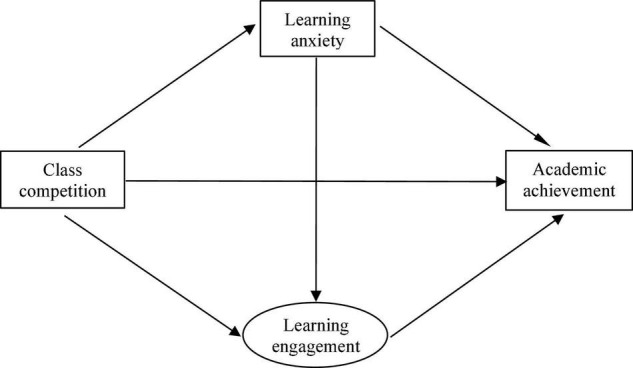
Hypothesis model of the relation between class competition and academic achievement.

Previous studies reported inconsistent findings about the relation between class competition and student academic achievement. We speculated that this may be related to the dual effects of class competition on students. On the one hand, class competition may have a negative effect on students’ academic achievement by increasing their learning anxiety; on the other hand, class competition may have a positive effect on their academic achievement by promoting learning engagement. However, few studies have explored this inconsistency and its underlying mechanisms, particularly in class competition among primary school students. Primary school students begin to face competition in the early stages of their learning careers, and investigating the underlying mechanisms is important for understanding the mode of action of class competition, promoting primary school students’ physical and mental health, and improving teaching outcomes. Therefore, the current study focused on primary school students to investigate the relation between class competition and academic achievement, as well as the mediating role of learning anxiety and learning engagement. Based on available research results, this study proposed the following hypotheses and a conceptual model (Figure 1).

**Hypothesis 1 (H1):** Class competition will have a direct effect on students’ academic achievement.
**Hypothesis 2 (H2):** Learning anxiety will play a mediating role between class competition and students’ academic achievement.
**Hypothesis 3 (H3):** Learning engagement will play a mediating role between class competition and students’ academic achievement.
**Hypothesis 4 (H4):** Learning anxiety and learning engagement will play multiple mediating roles between class competition and students’ academic achievement.

## Materials and Methods

### Participants

This survey was conducted in Hangzhou, Zhejiang Province, in Eastern China. We chose four primary schools (two from the city and two from rural areas) to reflect the overall situation of local schools. Considering the limitations of students’ psychological level and text understanding ability in grades one and two of primary school, we surveyed 40 classes from grade three to grade six in the four selected schools (10 classes for each grade). In total, 1,645 questionnaires were collected. After summarizing the questionnaire data and students’ exam results, and excluding omissions, overfilling, and missing results, data for 1,479 students were included in the analysis (effective rate 89.91%). Participating students were aged 8–13 years (*M* = 10.592 years; standard deviation = 1.204 years), 46.1% of them were girls, and 53.9% were boys.

### Measuring Instruments

#### Class Competition

Class competition is often regarded as a dimension of class environment. The most influential class environmental measurement scale has mainly been used to examine classes in schools in western countries (Fraser et al., 1982; Moos and Trickett, 1987). In the Chinese cultural context, classes in Chinese schools are very different to those in western countries. To ensure cultural applicability, this study chose the “My Class” scale compiled by Chinese researchers (Jiang, 2004). Previous studies have confirmed that this scale has good reliability and validity among Chinese students (Zhang et al., 2006). The scale consists of five dimensions, including teacher-student relationships, classmate relationships, order and discipline, class competition, and learning burden, with a total of 38 items. Among these dimensions, the class competition dimension consists of seven items (e.g., “Everyone is afraid of falling behind in learning” and “In order not to be surpassed by others, no one dares to relax in learning”). Responses are on a 5-point Likert-type scale from 1 (completely disagree) to 5 (completely agree), and the average score of the seven items was used as a student’s perceived class competition score. Higher scores indicate greater competition. The Cronbach’s alpha value for the class competition scale in this study was 0.682.

#### Learning Anxiety

The learning anxiety scale used in this study was derived from the Mental Health Diagnostic Test compiled by Zhou (1991), which has been widely used in thousands of primary and secondary schools in more than 20 provinces and cities in China, with high reliability and validity (Ding and Li, 2017; Hu et al., 2019). The learning anxiety subscale of the test scale has a total of 15 items (e.g., “When the teacher asks questions to the class, I feel uneasy about asking myself,” “I have dreamed about being reprimanded by parents or teachers because of my poor grades”). Responses were provided on a 5-point Likert-type scale from 1 (completely disagree) to 5 (completely agree), and the average score of the items was used as a student’s perceived learning anxiety score. Higher scores indicate greater learning anxiety. The Cronbach’s alpha value for the learning anxiety scale in this study was 0.887.

#### Learning Engagement

The Chinese version of the learning engagement scale in this study was translated and revised by Fang et al. (2008). The original scale was developed by Schaufeli et al. (2002a,b). The Chinese version of the scale has been reported to have good reliability and validity (Fang et al., 2008; Shi et al., 2013). The scale includes three dimensions and has a total of 17 items. Items 1–6 belong to the vitality dimension, items 7–11 to the dedication dimension, and items 12–17 to the concentration dimension. Examples of items are: “Even if my study is not smooth, I am not discouraged and can persevere” and “When I study, I forget everything around me.” Responses are provided on a 5-point Likert-type scale from 1 (completely disagree) to 5 (completely agree), and the average score of the 17 items was used as a student’s perceived learning engagement score. Higher scores indicate greater learning engagement. In this study, the three dimensions and the total scale had good reliability (vitality: Cronbach’s α = 0.849; dedication: Cronbach’s α = 0.861; concentration: Cronbach’s α = 0.881; total scale: Cronbach’s α = 0.938). The structural validity indicators were: χ^2^ (111) = 466.467, comparative fit index (CFI) = 0.975, Tucker–Lewis index (TLI) = 0.970, root mean square error of approximation (RMSEA) with 90% confidence interval (CI) = 0.047 (0.042–0.051), and standardized root mean squared residual (SRMR) = 0.024.

#### Academic Achievement

The indicators of academic achievement used in this study were based on four subjects: Chinese, mathematics, English, and science (full score for each course: 100 points). These four subjects are recognized as the main subjects in primary school in China and usually serve as an important reference to reflect the academic achievement of primary school students. In the current study, class competition refers to the overall feelings of students about the competitive atmosphere of the class, and not specifically to the feelings of competition in a certain subject. Therefore, the academic achievement of the students in this study also refers to the overall learning situation of the students, not that of a certain subject, so students’ academic achievement is calculated based on the average scores of the four subjects of Chinese, mathematics, English, and science. To ensure that the indicators of academic achievement were robust, we adopted the results of the last two final exams.

### Procedures

This study was first approved by the Ethics Committee of Hangzhou Normal University. We then applied to the administrative department for each selected school and obtained permission to conduct the study. Written informed consent was obtained from students’ parents before the investigation. After receiving consent, paper-and-pencil questionnaires were administered to students in class by trained graduate students who were majoring in psychology. It took approximately 20 min to complete the questionnaires. Students’ academic achievement data were provided by teachers at each school.

### Data Analysis

After the survey data were recovered, SPSS version 24.0 was used to test the valid sample data for missing completely at random (MCAR) data. The MCAR test results were: χ^2^ = 1254.895, df = 1206, *p* = 0.160 > 0.05, and the missing value was completely random. The Harman’s single-factor test was used to control the common method biases, and the first factor without rotation explained 24.44% of the variance, which was less than the critical value of 40%. Therefore, there were no obvious common method biases in this study. Descriptive statistics and correlations were calculated using SPSS version 24.0. A structural equation model and bias-corrected bootstrap test were then performed using Mplus version 7.0 to verify the mediating role of learning anxiety and learning engagement between class competition and academic achievement as well as to analyze the effect path.

## Results

### Descriptive Statistics and Correlation Analysis

The descriptive statistics and Pearson’s correlations among the variables are presented in Table 1. The average class competition score for primary school students was 3.16 points (range 1–5), indicating that the class competition atmosphere of primary school students was in a moderate level. In terms of relevance, class competition was significantly positively correlated with learning anxiety and learning engagement, learning engagement was significantly positively correlated with academic achievement, and learning anxiety was significantly negatively related to learning engagement and academic achievement. There was no significant correlation between class competition and academic achievement.

**TABLE 1:** Descriptive statistics and correlation analysis for each variable.

	1	2	3	4
1. Class competition	–			
2. Learning anxiety	0.205***	–		
3. Learning engagement	0.213***	-0.141***	–	
4. Academic achievement	−0.008	−0.190***	0.213***	
Range	1–5	1–5	1–5	0–100
Mean	3.16	3.25	3.52	83.52
Standard deviation	0.79	0.93	0.93	11.31
Male–female difference	5.018***	−3.092**	−0.130	−2.417*
Urban–rural difference	−3.905***	−4.735***	−1.641	4.519***

*p < 0.05, **p < 0.01, and ***p < 0.001.

Further analysis revealed that in addition to learning engagement, the three variables of class competition, learning anxiety, and academic achievement exhibited significant differences by gender and urban–rural dimensions. Regarding gender, the average class competition score of boys was significantly higher than that of girls, and the average learning anxiety and academic achievement scores of boys were significantly lower than those of girls. Regarding the urban–rural dimension, the average class competition and learning anxiety scores of urban students were lower than those of rural students, but the average academic achievement score of urban students was significantly higher than that of rural students.

### Mediating Effect Analysis of Learning Anxiety and Learning Engagement

As mentioned earlier, the results revealed no significant correlation between class competition and academic achievement. This finding raises the question of whether or not class competition has an effect on academic achievement. Some methodologists believe that whether independent variables and dependent variables are significantly correlated does not constitute a prerequisite for the existence of mediating effects (Collins et al., 1998; Shrout and Bolger, 2002; Preacher et al., 2007). A simulation study by Rucker et al. (2011) revealed that in nearly half of all simulation conditions, the relation between the independent variable and the dependent variable was not significant, but there was a significant mediating effect. Therefore, it is necessary to further analyze mediating effects despite the lack of a significant correlation between students’ perceived class competition and their academic achievement.

**FIGURE 2:**
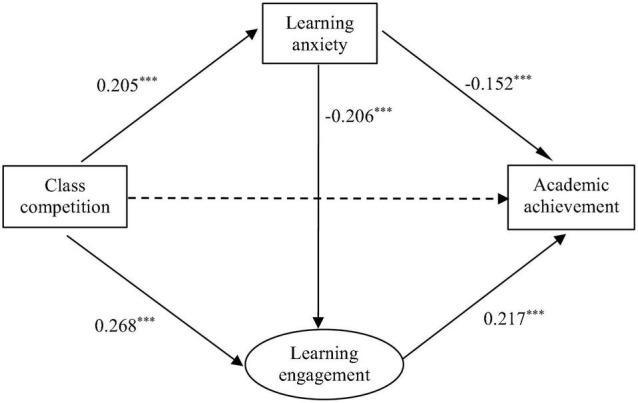
Mediation effect model of class competition on academic achievement. ***p < 0.001.

The starting theoretical model (Figure 1) showed a good fit to the empirical data: χ^2^(6) = 29.135, CFI = 0.992, TLI = 0.981, RMSEA (90% CI) = 0.051 (0.033–0.070), and SRMR = 0.014. The results indicated that the effect of class competition on academic achievement was not significant, but learning anxiety and learning engagement fully mediated the relation between class competition and academic achievement (Figure 2).

Finally, we used a bias-corrected bootstrap method to test whether the mediating effects described above were significant. In general, if the 95% CI does not include 0, the mediating effect is considered to be significant at the 0.05 level (Preacher and Hayes, 2008). The results revealed that the mediating effects of learning anxiety and learning engagement were significant, and class competition affected academic achievement through the three possible paths: class competition had a negative effect on academic achievement through increasing learning anxiety, a positive effect on academic achievement through promoting learning engagement, and a chain effect on academic achievement through learning anxiety *via* learning engagement (Table 2).

**TABLE 2:** Bias-corrected bootstrap test on mediating effects.

Paths	Standardized estimates	95% confidence interval	
		Lower	Upper
Class competition-academic achievement	−0.025	−0.080	0.028
Class competition-learning anxiety-academic achievement	−0.031***	−0.047	−0.018
Class competition-learning engagement-academic achievement	0.058***	0.040	0.078
Class competition-learning anxiety-learning engagement-academic achievement.	−0.009***	−0.014	−0.006

***p < 0.001.

## Discussion

### The Current Situation of Main Variables

The results in Table 1 show that the average class competition score of primary school students in the current study was 3.16 points (score range: 1–5), indicating that primary school classes had a clear competitive atmosphere. In China, many parents expect their children to be among the best in class rankings (Deng and Zou, 2015). Students with advantages in class competition are often favored by teachers and tend to have greater influence in the student group, while students with disadvantages in class competition have difficulty in becoming influential figures in the class (Warren et al., 2005). According to the relative deprivation theory (Davis, 1966), individuals’ sense of relative deprivation arises from comparison with others, and their sense of competition will be stimulated when perceiving inferior position. Given the combined effects of pressure from parents, teachers and other factors, primary school students are increasingly likely to experience Competition (Liu et al., 2020).

Regarding the gender differences in class competition, learning anxiety, and academic achievement among primary school students revealed in the current results, we believe that, in addition to boys being more sensitive to the competitive atmosphere (Volk et al., 2006), the high familial expectations in traditional Chinese culture concerning the academic achievement and future careers of male children in particular may also increase boys’ sense of competitive pressure. This is consistent with the conclusion of a previous study that boys’ perception of the class competition atmosphere is higher than that of girls (Li et al., 2019). Emotion-related characteristics of girls (Pekrun and Stephens, 2010) may explain why girls’ learning anxiety was higher than that of boys in the current results. Boys have been reported to exhibit significantly lower levels of school adaptation and lower learning consciousness compared with girls (Zhang et al., 2014; Liu et al., 2020), which may explain why the academic achievement of girls in primary school is higher than that of boys.

The differences between urban and rural students in class competition, learning anxiety and academic achievement may be related to the imbalance between urban and rural education in China. Compared with urban students, rural students have fewer employment channels, and changing their employment trajectory through studying hard is often the only option for rural children’s future development. As Pettigrew et al. (2008) pointed out, individuals’ socioeconomic status significantly affects their sense of relative deprivation, and those with lower social status and less political influence experience stronger sense of relative deprivation, and strive to change the *status quo*. Compared with urban students, rural students have a stronger sense of relative deprivation, which further intensifies competition in class and increases their learning anxiety. However, because the quality of teachers and teaching facilities in rural schools lags behind that in urban areas, the education received by rural students is significantly worse than that of urban students (Sang et al., 2009). This reality may be the main reason why rural students’ academic achievement is not as good as that of urban students.

### Direct Effect of Class Competition on Academic Achievement

This study investigated the mechanism of the effect of class competition on academic achievement among primary school students by establishing a multiple mediating model for the first time. The results revealed that class competition had no significant direct effect on students’ academic achievement, which failed to support H1. A previous study reported that class competition had different effects on students with different levels of academic achievement, such that students with good grades felt the pressure brought by competition, leading them to study harder and achieve better grades, whereas students with poor grades lacked self-confidence, causing their learning enthusiasm and motivation to be further decreased by repeated failures in class competition, leading to worse grades (Zimmerman, 2003). The polarization of academic achievement caused by class competition counteracts the effect of class competition on academic achievement to some degree, which may explain the absence of a significant correlation between these two variables in the current study.

The results of this study were inconsistent with the findings of many previous studies (Ames and Archer, 1988; Sommet et al., 2013). We believe that these discrepancies may have arisen because most previous research focused on students from junior high schools, senior high schools, and universities, whereas we investigated primary school students. Students’ perception of the classroom psychological environment is reported to vary across education stages (Zhang et al., 2014), and their academic achievement is the result of interactions among many factors, including their family background and school environment (Feng et al., 2019). Therefore, it is understandable that the relation between class competition and academic achievement in this study differed from that reported in previous studies.

### Mediating Roles of Learning Anxiety and Learning Engagement

The current findings indicate that class competition can be a “double-edged sword” for academic achievement through the intermediating variables of learning anxiety and learning engagement. These findings supported H2 and H3. Class competition negatively affected students’ academic achievement by increasing their learning anxiety, which was consistent with conclusions drawn by Sariem et al. (2014) and D’Agostino et al. (2021). According to Lewin’s field theory (Lewin, 1936), individuals’ mental activity and behaviors are closely related with the environment they live. For students who daily live in a competitive atmosphere, the pressure of comparison and potential failures leads them to feel uneasy and anxious about learning outcomes. Anxiety is experienced particularly strongly when control over the outcome of a competition seems impossible (Pekrun, 2006). The conclusion that class competition can negatively affect students’ academic achievement by increasing their learning anxiety may serve as a reminder for teachers and parents to pay attention to the learning-related emotions of primary school students. Reducing class competition and alleviating students’ learning anxiety *via* home–school cooperation may enable students to study in a relaxed and happy state, thereby improving their learning outcomes.

Conversely, the current results also revealed that class competition positively affected students’ academic achievement by promoting their learning engagement, which was consistent with the results of several previous studies (Roth et al., 2009; Reyes et al., 2012; Bayoumy and Alsayed, 2021). The pressure of class competition means that students who want to satisfy their needs for academic achievement or class status have to increase their learning engagement by using certain strategies (e.g., listening carefully in class and reviewing spontaneously after class) to achieve better grades. One study found no significant correlation between learning engagement and academic achievement (Shernoff, 2010). We propose that this finding may have arisen because students with good grades master fast learning skills, thereby reducing their learning time. In contrast, students with poor grades do not have a good foundation for those skills, and it remains difficult for them to achieve good grades even if they struggle and invest more time (Phuntsho and Dhendup, 2020). This highlights the need for teachers to pay attention to students’ learning foundations, learning style, and learning habits, and to guide students to adopt a learning method that suits their specific characteristics. Furthermore, Schaufeli et al. (2002b) divided learning engagement into three dimensions (vitality, dedication, and concentration). In the current study, we did not perform comprehensive analyses of the specific roles of these three dimensions in the relation between class competition and academic achievement. This question should be addressed in future research.

The current study revealed that class competition negatively affected students’ academic achievement through multiple mediating effects of learning anxiety and learning engagement. This finding supported H4 and also revealed another possible path by which class competition indirectly affected academic achievement (i.e., class competition positively affected students’ learning anxiety, learning anxiety negatively affected learning engagement, and learning engagement positively predicted academic achievement). Therefore, the effects of class competition on academic achievement may be determined by learning anxiety and learning engagement. This conclusion further demonstrated the close relation between learning anxiety and learning engagement. As Kindermann (1993) noted, learning engagement is essentially students’ emotional engagement in learning activities. The control value theory proposed by Pekrun (2006) also details this process: emotions can affect students’ academic achievement by influencing their motivation and effort, their use of learning strategies and self-regulation, and the availability of cognitive resources needed for learning and performance. Some researchers in the field of psychology verified the impact of learning anxiety on personal values and individual behavioral input through empirical research and follow-up surveys (Gao et al., 2021). Class competition can affect academic achievement through multiple mediating effects of learning anxiety and learning engagement, which reflects the chain relation among the four variables and demonstrates another way in which learning anxiety and learning engagement play a mediating role between class competition and academic achievement.

### Limitations and Contributions

Several limitations of the current study should be acknowledged. First, the sample was selected from four primary schools in Hangzhou, Zhejiang Province, China. However, the educational and cultural contexts in China differ from those in other countries, and further research should examine a wider range of samples to include students in different cultural contexts. Second, this study mainly focused on individual students’ perceptions of the competitive atmosphere in class. However, classes with different levels of competition may have different effects on students. Therefore, in future studies, multilevel modeling should be adopted to model class-level effects. Third, this study focused on the relation between students’ overall feelings about class competition and students’ learning anxiety, learning engagement and academic achievement, which are not specific to individual subjects. However, the relations between the four variables may vary between different subjects. Thus, relevant research for specific disciplines should be carried out in future research. Fourth, although the reliability of the class competition scale in this study was acceptable, it is still low, and the survey process should be further improved. In addition, because this was a cross-sectional study, the causal direction of the hypothesized effects should be confirmed in longitudinal research.

Despite these limitations, the current study has important theoretical implications and practical value. From a theoretical perspective, this study expanded the research on competition among primary school students and also provides a theoretical explanation for the inconsistent effects of class competition on academic achievement. From a practical perspective, the existence of class competition is inevitable. The results of this study can guide teachers in taking effective measures to enable students to strengthen their learning engagement levels actively in an atmosphere of benign competition. At the same time, our findings suggest that teachers should pay close attention to students’ psychological states and create cooperative classroom learning environments at an appropriate stage to give students appropriate encouragement, thereby reducing the negative effects of class competition and avoiding learning anxiety (Okebukola, 1986). In addition, teachers should guide students to improve their learning methods and form good learning habits, thereby improving learning engagement and promoting a high level of academic achievement.

## Conclusion

The current study focused on primary school students to investigate the relation between class competition and academic achievement, as well as the mediating roles of learning anxiety and learning engagement. The following three findings were highlighted. First, the class competition atmosphere of primary school students we investigated was in a moderate level. Except learning engagement, class competition, learning anxiety, and academic achievement exhibited significant differences in gender and urban–rural residence. Second, class competition did not have a direct relation with academic achievement. Third, class competition was significantly negatively correlated with academic achievement *via* learning anxiety, whereas was positively correlated with academic achievement *via* learning engagement. Besides, class competition was negatively associated with academic achievement *via* the multiple mediating roles of learning anxiety and learning engagement.

## Data Availability Statement

The raw data supporting the conclusions of this article will be made available by the authors, without undue reservation.

## Ethics Statement

The studies involving human participants were reviewed and approved by the Academic Ethics Committee of Jing Hengyi School of Education, Hangzhou Normal University. Written informed consent to participate in this study was provided by the participants’ legal guardian/next of kin.

## Author Contributions

GL and RZ developed the research project, with the contribution of XW and ZL. ZL prepared the data set. XW carried out the data analysis. ZL reviewed the literature. GL and RZ reviewed and edited the manuscript. All authors contributed to the article and approved the submitted version.

## Conflict of Interest

The authors declare that the research was conducted in the absence of any commercial or financial relationships that could be construed as a potential conflict of interest.

## Publisher’s Note

All claims expressed in this article are solely those of the authors and do not necessarily represent those of their affiliated organizations, or those of the publisher, the editors and the reviewers. Any product that may be evaluated in this article, or claim that may be made by its manufacturer, is not guaranteed or endorsed by the publisher.
